# Notes and News

**Published:** 1899-08-19

**Authors:** 


### NOTES AND NEWS.

(Collected and Communicated.)

Miss Hankey, of Walmer Beach, has presented a new wing to the Victoria Cottage Hospital at Deal, and it has now been opened.

An enlightened spirit in regard to the treatment of their dispensary doctors is not yet abroad in Ireland. Not only do most Boards of Guardians show a disposition to reduce instead of increasing salaries, but holidays are being curtailed also in the face of recommendations of the Local Government Board.

Special efforts must be made by those interested in the establishment of the new infirmary at Newcastle to secure the £100,000 bequeathed for the purpose. The gift is conditional on the raising of £100,000 by the public to maintain the infirmary as a local Jubilee offering. Also the subscriptions must be secured within three years of the bequest being made. £10,000 is still wanting to ensure the gift.

At the last meeting of the British Laryngological, Rhinological, and Otological Association the following officers for the ensuing year were elected: President, Dr. Barclay Baron; vice-presidents, Dr. Percy Jakins, Mr. John Bark, and Mr. Wyatt Wingrave; treasurer, Dr. Dundas Grant; honorary secretaries, Mr. St. George Reid and Dr. Chichale Nourse. Among the various cases described to the meeting was one by Dr. Furniss Potter, in which recovery had taken place, under the use of iodides for nearly six months, after complete recurrent paralysis of the left vocal cord, together with marked paresis of the left side of the soft palate and of the tongue.

Sir J. G. Rogers (Pasha), Director-General Sanitary Department, Egypt, has sent to the *Times* a long criticism of Haffkine's proposals in regard to inoculation. He points out that even supposing there was a sufficient supply of the material at hand, and that a sufficient number of skilled operators were available to ensure the inoculation of 10,000 persons a day, it would have taken 32 days to protect Alexandria, whereas by the sanitary measures adopted, although the disease may not have been entirely stopped, at least this result has been secured, that "in 100 houses in which plague cases or suspicious cases occurred, and which were efficiently disinfected, in not one instance has a second case been noted."

Mr. Jonathan Hutchinson has presented his native town, Selby, with a museum. The building was originally used as public rooms until Mr. Hutchinson bought it and fitted it up for the purpose of disseminating scientific knowledge. Mr. Hutchinson has himself held lectures at the museum.

The British and American Church in St. Petersburg has succeeded in exciting a great deal of interest and sympathy for the poor of Russia in the famine-stricken districts. Large sums have been sent from England; and Mr. Woolrych Perowne succeeded in collecting £500, to which the readers of The Hospital contributed. Five English ladies have devoted themselves to distributing relief, acting with the Red Cross and local authorities.

There has lately been an outbreak of diphtheria in the Willard State Hospital in the United States. Thirty-four cases have been reported, and a sort of quarantine has been established. What is of interest is that bacteriological examination showed that many of the physicians, nurses, servants, and others connected with the management of the institution had diphtheria bacilli in the mucous membranes of their mouths and upper air passages. This accords with what has been found to be the case in many outbreaks of the disease.

Still another unpaid medical service is to be extracted from the medical profession. The Chief City Health Commissioner of Chicago has issued a call, says the *Medical News* of New York, for volunteer physicians to serve as medical inspectors, and more than 40 have been enrolled upon the books of the department, to undertake a house-to-house canvass for the purpose of disseminating knowledge as to the care of infants and to inspect sanitary conditions. The object is in the first place to instruct mothers and those in charge of infants, during the hot weather, as regards hygiene of the young, their feeding, &c., with the view to the prevention rather than the cure of disease; and, secondly, to correct sanitary defects and abate nuisances prejudicial to the health of adults and children alike.

In the United States we seem to meet with the extremes of civilisation and barbarism. A week or two ago a Dr. Froelich was brutally assaulted by some motor men engaged in a New York tramway strike, and all because he had dressed the wounds of a non-striker, who had previously suffered at their hands. It appears, according to the account given in the New York *Medical Record*, that he was told by a passer-by that a man with his head cut was in the next block, and on going there he found a man in the uniform of a motorman with a long gash in his scalp. He stopped the bleeding and dressed the wound as best the could for the time, and while so engaged he was warned by one of the crowd of strikers that he had better leave the man alone if he valued his own health. He finished dressing the wound and started on his way home, but had not gone far before he was struck on the head with a brick, and was rushed upon by eight men, who beat him severely before the police arrived. It would seem as if we wanted a Geneva Convention in regard to our social wars and trade disputes as much as in wars between one nation and another. Nowhere are strikes attended with such violence and outrageous reversion to pristine barbarity as in the United States, where no respect is paid even to the wounded.

